# Tranexamic Acid; A Glittering Player in the Field of Trauma 

**DOI:** 10.30476/BEAT.2020.46443

**Published:** 2020-04

**Authors:** Fariborz Ghaffarpasand, Hamid Reza Abbasi, Shahram Bolandparvaz, Shahram Paydar, Maryam Dehghankhalili

**Affiliations:** 1 *Research Center for Neuromodulation and Pain, Shiraz University of Medical Sciences, Shiraz, Iran*; 2 *Trauma Research Center, Shiraz University of Medical Sciences, Shiraz, Iran*; 3 *Student Research Committee, Shiraz University of Medical Sciences, Shiraz, Iran*

**Keywords:** Tranexamic Acid, Trauma, Coagulopathy, Antifibrinolytics, Hemorrhage

Trauma is still the leading cause of mortality and morbidity worldwide with an estimated 5.8 million mortalities every year [1] and approximately 60 million traumatic brain injuries (TBI) annually [2]. Hemorrhage remains the most common preventable cause of mortality and morbidity following trauma either in civilian or military settings [3, 4]. Intracranial bleeding following TBI results in increased intracranial pressure (ICP), brain herniation and cerebral edema which are all secondary insults to the brain parenchyma leading to increase disability and mortality [5]. Thus, the development and administration of antifibrinolytic agents have been the focus of traumatic injuries during the previous decades with the hypothesis of hemorrhage cessation and hemostasis with a medical agent rather than a surgical intervention. These efforts resulted in developing several agents and subsequent large multicenter clinical trials to define the best antifibrinolytic agent for prevention of death following TBI. 

Tranexamic acid (TXA), an antifibrinolytic agent being introduced in 1962, has been spotlight of the TBI treatment during the past decade [6]. TXA provides its antifibrinolytic effects through binding to the plasminogen molecule which in turns blocks connection of the plasminogen to the plasmin and fibrin. These cascades lead to stabilization of the formed network through secondary hemostasis. The drug is administered through oral and intravenous routes and has a bioavailability of 33 and 90% respectively [7]. Several applications have been approved for the TXA including the trauma, obstetrics and gynecology condition (menstrual bleeding, obstetrics bleeding), orthopedics surgery, spinal surgeries, dental procedures, hemoptysis, hemophilia, and epistaxis [6, 8, 9]. 

Until now, several studies have addressed the effects of the TXA on the patients with trauma with an emphasis on the TBI [10-12]. The two main projects accordingly include the Clinical Randomization of an Antifibrinolytic in Significant Hemorrhage (CRASH) [10, 11] and Military Application of Tranexamic Acid in Trauma Emergency Resuscitation (MATTERs) [13]. Very recently, the results of the CRASH-3 was published which provides the highest level of evidence regarding the efficacy and safety of the TXA in patients with TBI [10]. In addition, several lines of recent evidence have demonstrated that pre-hospital and early administration of TXA leads to clot stabilization and a reduction of fibrinolytic activity, causing a decrease in fibrin degradation products buildup (D-dimer) [14] which in turn is associated with prolonged time to death and significantly improved early survival [15]. 

We herein, discuss and summarize the results of these three main studies in order to emphasis on the results and utilize them in the current clinical practice. Morrison et al. [13] in MATTRs study investigates the effects of TXA administration on outcome of combat injuries. They have included a total number of 896 consecutive admissions with combat injury in 3 centers in southern Afghanestan, of which 293 received TXA. They demonstrated that the use of TXA with blood component-based resuscitation following combat injury results in improved measures of coagulopathy and survival, a benefit that is most prominent in patients requiring massive transfusion [13]. In CRASH-2 which was a randomized clinical trial (RCT), 10 hospitals with 270 participants were enrolled. Participants were randomly allocated to receive either a loading dose of 1 g of TXA infused over 10 minutes followed by an intravenous infusion of 1 g over 8 hours or matching placebo. The results reported that neither moderate benefits nor moderate harmful effects can be excluded. However, the secondary analyses demonstrated that the TXA might have beneficial effects for those with TBI which required larger studies with more participants [11]. Thus, the study groups developed and started the CRASH-3 study which was recently published in the Lancet [10]. In this recently published RCT, 175 hospitals in 29 countries with 12737 patients with TBI were enrolled. Patients were randomly assigned to receive TXA (loading dose 1 g over 10 min then infusion of 1 g over 8 h) or matching placebo. The results clearly demonstrated that TXA is safe in patients with TBI and that treatment within 3 hours of injury reduces head injury-related death. Patients should be treated as soon as possible after injury with TXA [10].

After 2 decades of research on TXA in trauma, the latest lines of evidence clearly demonstrate that the early administration after TBI is associated with both decreased trauma associated mortality and morbidity. The important point that should be taken in mind is the timing of the administration. As there is a direct relationship between the time of administration and the mortality and morbidity following TBI, it is recommended that TXA be administered as soon as possible and even in the pre-hospital setting. General studies have also clearly demonstrated that pre-hospital and early administration of TXA in trauma patients is associated with decreased mortality. Currently, some studies are underway to investigate the efficacy of TXA administration on outcome of trauma patients in pre-hospital setting. Although, research is ongoing in the field, however, it seems that TXA efficacy in decreasing mortality and morbidity in trauma patients is confirmed by now and the TXA is in the middle of the spotlight of trauma treatment. 

## Conflict of Interest:

None 
